# Caput Ligation Renders Immature Mouse Sperm Motile and Capable to Undergo cAMP-Dependent Phosphorylation

**DOI:** 10.3390/ijms221910241

**Published:** 2021-09-23

**Authors:** Darya A. Tourzani, Maria A. Battistone, Ana M. Salicioni, Sylvie Breton, Pablo E. Visconti, Maria G. Gervasi

**Affiliations:** 1 Department of Veterinary and Animal Sciences, Integrated Science Building, University of Massachusetts, Amherst, MA 01003, USA; dtourzan@umass.edu (D.A.T.); asalicioni@vasci.umass.edu (A.M.S.); 2 Program in Membrane Biology, Nephrology Division, Massachusetts General Hospital and Harvard Medical School, Boston, MA 02115, USA; mbattistone@mgh.harvard.edu (M.A.B.); breton.sylvie@mgh.harvard.edu (S.B.)

**Keywords:** epididymal ligation, epididymis, sperm, caput, cauda, motility, fusion, post-translational modifications

## Abstract

Mammalian sperm must undergo two post-testicular processes to become fertilization-competent: maturation in the male epididymis and capacitation in the female reproductive tract. While caput epididymal sperm are unable to move and have not yet acquired fertilization potential, sperm in the cauda epididymis have completed their maturation, can move actively, and have gained the ability to undergo capacitation in the female tract or in vitro. Due to the impossibility of mimicking sperm maturation in vitro, the molecular pathways underlying this process remain largely unknown. We aimed to investigate the use of caput epididymal ligation as a tool for the study of sperm maturation in mice. Our results indicate that after seven days of ligation, caput sperm gained motility and underwent molecular changes comparable with those observed for cauda mature sperm. Moreover, ligated caput sperm were able to activate pathways related to sperm capacitation. Despite these changes, ligated caput sperm were unable to fertilize in vitro. Our results suggest that transit through the epididymis is not required for the acquisition of motility and some capacitation-associated signaling but is essential for full epididymal maturation. Caput epididymal ligation is a useful tool for the study of the molecular pathways involved in the acquisition of sperm motility during maturation.

## 1. Introduction

After leaving the testis, sperm require two additional post-testicular maturational processes to be able to fertilize, one in the male reproductive tract known as epididymal maturation [1,2,3,4], and the second one in the female reproductive tract known as capacitation [5,6]. Once leaving the testis, the immature sperm enter into the epididymis, an extended convoluted tubule that is compartmentalized into several regions [7,8]. In the mouse epididymis, from proximal to distal, four major anatomical regions can be differentiated: initial segment, caput, corpus and cauda [9]. These regions present different histological characteristics as well as specific luminal content [10]. Immature sperm found in the initial segment and in the caput epididymis are immotile when suspended in physiological solutions [1,3,4]. Once in the cauda, released mature sperm are progressively motile and have acquired the ability to capacitate in the female reproductive tract and also in vitro [6]. Capacitation is associated with the activation of several signaling pathways including but not limited to an increase in cAMP levels with the consequent activation of a protein phosphorylation cascade. Importantly, in immature sperm, phosphorylation is impaired even in the presence of exogenous permeable cAMP analogues [11,12].

As sperm are transcriptionally and translationally silent cells [13], epididymal maturation has been proposed to involve acquisition of new molecules either directly by endocytosis or, more likely, by fusion with epididymal exosomes known as epididymosomes [14,15]. Epididymosomes contain proteins, non-coding RNAs, lipids and metabolites that incorporate into sperm cells and are hypothesized to contribute to signaling [16,17] and epigenetic [18,19] pathways. In addition to new molecules, it is clear that maturation requires post-translational modifications of sperm proteins. In particular, epididymal maturation is associated with inactivation of the serine/threonine protein phosphatase 1 gamma Ppp1cc (also known as PP1γ2) [20,21]. More recently, our group has shown that protein O-GlcNAcylation changes in sperm during epididymal maturation [12]. Immature caput sperm exhibited high O-Linked β-N-Acetylglucosamine (O-GlcNAc) Transferase (OGT) protein levels corresponding with high levels of protein O-GlcNAcylation while mature cauda sperm exhibited levels of OGT and O-GlcNAcylation significantly lower [12]. The relation between O-GlcNAcylation and phosphorylation pathways has been documented in somatic cells [22,23,24]. Then, we hypothesized that the interplay between O-GlcNAcylation and protein phosphorylation during epididymal maturation is key for the sperm cell to acquire motility, to activate capacitation-induced signaling pathways (e.g., phosphorylation) and, most importantly, to gain fertilizing capacity.

Early reports indicate that restricting the passage of sperm from caput to corpus and cauda by surgical ligation is sufficient to induce sperm motility in a variety of model species [25,26,27,28]. However, little is known on how surgical isolation affects caput sperm signaling pathways and other functional parameters. In this work, we used a mouse model to investigate the extent by which epididymal ligation changes sperm functional parameters including motility patterns, fertilizing capacity and the ability to fuse with zona-pellucida-free metaphase II-arrested oocytes. In addition, we tested the effect of ligation on phosphorylation and O-GlcNAcylation. Our results indicate that by the application of caput epididymal ligation sperm undergo molecular and physiological changes related to sperm maturation and suggest that this method can be used as a tool for the study of sperm maturation processes in mice.

## 2. Results

### 2.1. Epididymal Ligation Induces Motility in Immature Caput Mouse Sperm

**Figure 1:**
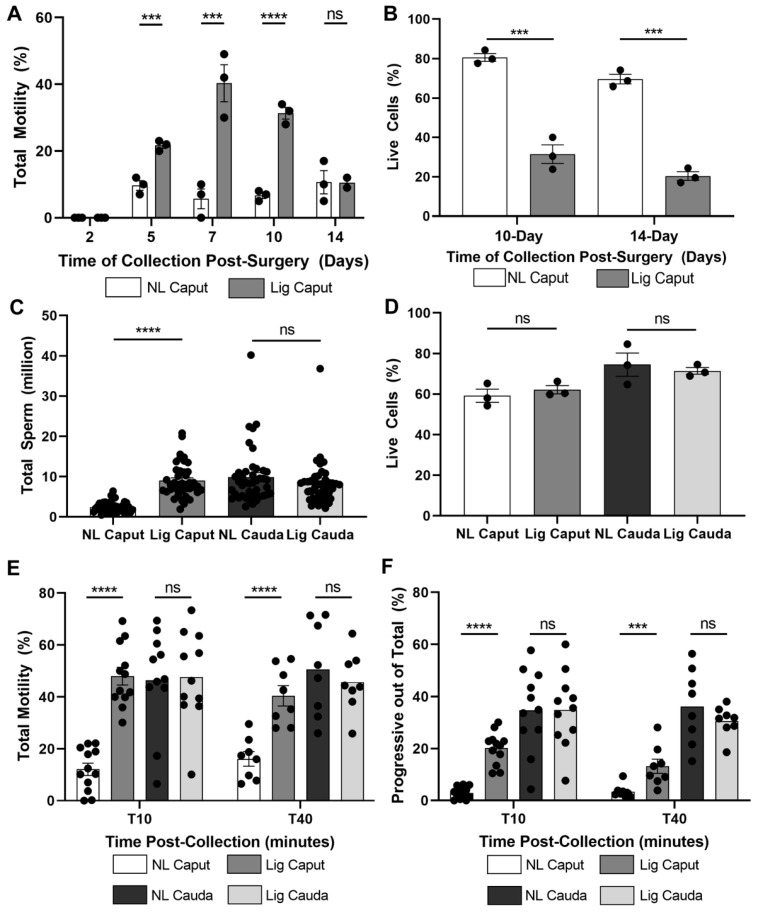
Ligation of the epididymis in the distal caput region induces motility of immature sperm. Comparison of sperm samples collected from the non-ligated caput (NL Caput) region and ligated caput (Lig Caput) region. Sperm samples were incubated in mTYH-capacitating medium supplemented with HCO_3_^−^ (25 mM) and BSA (5 mg/mL). Motility was assessed using Computer Assisted Sperm Analysis (CASA). (**A**) Total motility was compared between sperm recovered from the NL Caput and Lig Caput at 2-, 5-, 7-, 10-, and 14-days post-ligation surgery. Total motility was assessed after 10 min in capacitation medium. *n* = 3. (**B**) Sperm viability from non-ligated caput and ligated caput at 10-days and 14-days post ligation surgery was evaluated with a live–dead assay. *n* = 3. (**C**) Total sperm recovered from each experimental condition at 7-days post-ligation surgery. *n* = 42. (**D**) Sperm viability from each experimental condition at 7-days post-ligation surgery was assessed with a live–dead assay. *n* = 3. (**E**) Total motility during capacitation was further assessed at 10- and 40-min of capacitation in sperm recovered from each experimental condition. T10 *n* = 12, T40 *n* = 8. (**F**) Progressive motility out of the total sperm population was analyzed at 10- and 40-min of capacitation in sperm recovered from each experimental condition. T10 *n* = 12, T40 *n* = 8. Statistical significance comparing NL Caput vs. Lig Caput and NL Cauda vs. Lig Cauda using Sidak–Bonferroni *t*-test in all graphs indicated; ns *p* > 0.05, *** *p* < 0.001, **** *p* < 0.0001.

To test the effect of caput ligation on motility, we collected sperm from caput and cauda epididymis from both the non-ligated epididymis (control) and the ligated epididymis (experimental) of the same animal (as shown in Figure S1). Sperm were suspended in capacitating media containing HCO_3_^−^ and bovine serum albumin (BSA) and the percentage of motile cells was analyzed by Computer Assisted Sperm Analysis (CASA) at different days post-surgery (Figure 1A). We found that motility increased with time, reaching a maximum at 7 days and then decreased; this decrease was correlated with lower sperm viability (Figure 1B). Therefore, we choose 7 days post-surgery for the following experiments. As ligation generates a barrier preventing sperm transit to the distal regions of the epididymis, we evaluated the number of sperm recovered. As expected, the ligated caput has significantly more sperm than the non-ligated caput (Figure 1C). However, no differences were found in the cauda region (Figure 1C). Importantly, no differences between ligated and non-ligated caput or cauda sperm viability were observed 7 days post-ligation (Figure 1D). We then evaluated the effect of ligation on total and progressive motility under capacitating conditions. The overall motility of ligated caput sperm was greater compared to control caput sperm and remained high for up to 40 min of incubation (Figure 1E). In addition, ligated caput sperm had higher percentage of progressive motility than non-ligated caput sperm after 10 and 40 min of capacitation (Figure 1F). Contrary to cauda sperm, ligated caput sperm did not hyperactivate (data not shown). Together, these results indicate that, similar to other species, epididymal ligation of the distal caput region can induce motility of immature caput sperm in mice.

### 2.2. Epididymal Ligation Produces Enlargement of Caput Epididymal Tubule and Affects Expression of Aquaporin-9 in Principal Cells

**Figure 2:**
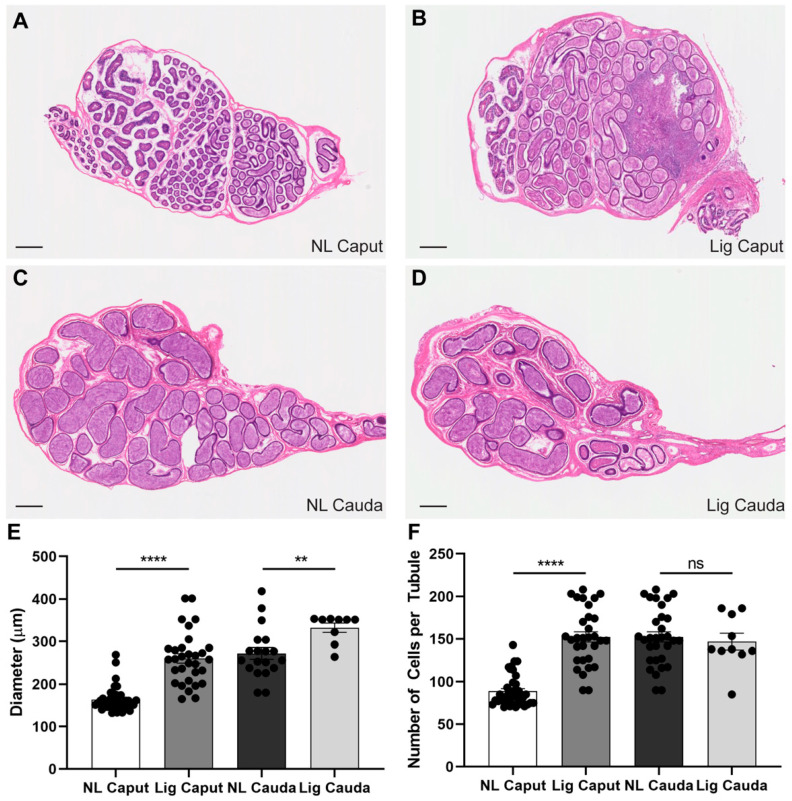
Effects of ligation on epididymal tissues. H&E histological staining comparison of the four epididymal experimental regions at 7-days post ligation surgery. (**A**–**D**) Sections of non-ligated caput (NL Caput), ligated caput (Lig Caput), non-ligated cauda (NL Cauda) and ligated cauda (Lig Cauda) regions. Scale bar = 200 µm. *n* = 3. (**E**) Quantification of tubule diameter comparison between the four experimental regions. Independent experiments *n* = 3; NL and Lig Caput tubules *n* = 40, NL and Lig Cauda tubules *n* = 10. (**F**) Quantification of Hoechst-positively stained cells comparing the number of cells per round tubule. Independent experiments *n* = 3; NL and Lig Caput tubules *n* = 40, NL and Lig Cauda tubules *n* = 10. Statistical significance comparing NL Caput vs. Lig Caput and NL Cauda vs. Lig Cauda using an unpaired *t*-test in all graphs indicated; ns *p* > 0.05, ** *p* < 0.01, **** *p* < 0.0001.

Due to the nature of the method causing an obstruction in the epididymis, we investigated potential changes in the epithelium of this organ at 7 days post-surgery. We conducted histological studies of the caput and cauda of both the ligated and non-ligated sides using hematoxylin and eosin staining (H&E) (Figure 2). Ligated caput epididymides presented a large portion of tissue damaged and enlarged tubules when compared to non-ligated caput sections (Figure 2A,B). Overall, ligated cauda epididymides exhibited normal morphology; however, we observed slightly larger tubules when compared to non-ligated cauda (Figure 2C,D). These morphological observations were quantified by measuring the diameter of the tubules (Figure 2E) as well as the number of cells in the lining epithelium of the tubules (Figure 2F). 

**Figure 3:**
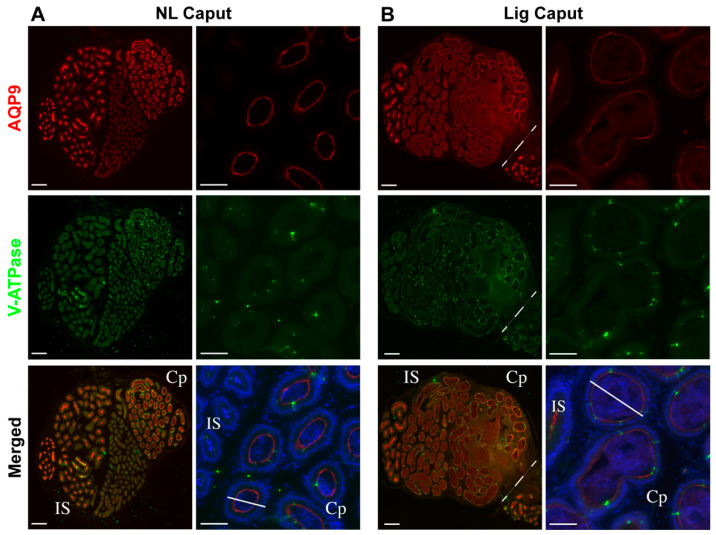
Expression of epididymal cell type markers after ligation. Confocal microscopy of tissue sections from the NL Caput and Lig Caput regions at 7-days post-ligation surgery. (**A**) NL Caput tissue sections were co-stained with AQP9 (red) to mark principal cells (upper panel), V-ATPase (green) to mark clear cells (middle panel), and DAPI (blue, merged with AQP9 and V-ATPase, lower panel). Scale bars: 100 µm (main images, left); 50 µm (magnifications, right). *n* = 3. (**B**) Lig Caput tissue sections were co-stained with AQP9 (red) to mark principal cells (upper panel), V-ATPase (green) to mark clear cells (middle panel), and DAPI (blue, merged with AQP9 and V-ATPase, lower panel). Scale bars: 100 µm (main images, left); 50 µm (magnifications, right). *n* = 3. The ligation suture region is indicated by a dotted line in the Lig Caput sections. Localization of specific epididymal regions were identified: initial segment (IS) and caput (Cp). White lines across the tubules are representative of the tubule diameter measurements.

The potential changes of the epididymal cell composition were evaluated by immunofluorescence of key epididymal cell markers. Sections of each region were stained with antibodies that detect V-ATPase (marker of clear cells) and aquaporin-9 (AQP9, marker of principal cells). The localization of AQP9 was markedly reduced in the ligated initial segment and caput when compared to the non-ligated side (Figure 3A,B). Though, the localization of V-ATPase in ligated and non-ligated caput was similar (Figure 3A,B). Additionally, although no differences were found when comparing the localization of these markers in the cauda region, we observed absence of rows of clear cells in the cauda (Figure S2A,B).

### 2.3. Ligated Caput Sperm Are Able to Activate cAMP-Dependent Phosphorylation When Incubated in Capacitation-Supporting Media

**Figure 4:**
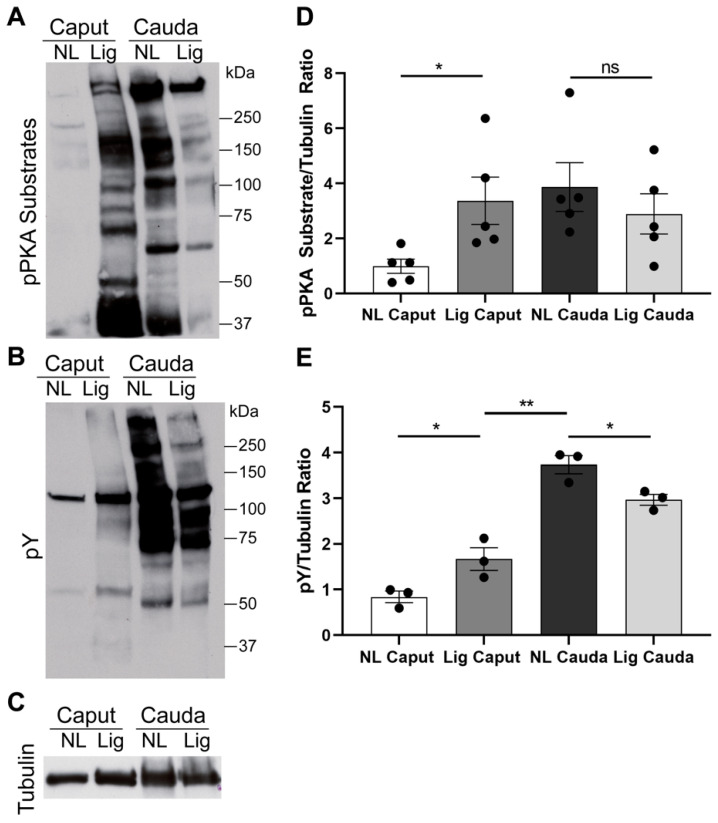
Changes to post-translational modifications in sperm after epididymal ligation. Sperm samples were collected from each experimental region 7-days post-ligation surgery: NL Caput, Lig Caput, NL Cauda, and Lig Cauda. Samples were capacitated for 40 min prior to protein extraction and separation by SDS-PAGE. (**A**) Western blot of phosphorylated protein kinase A substrates (pPKA substrates). *n* = 5. (**B**) Membranes were stripped and re-probed with an antibody against tyrosine phosphorylation (pY). *n* = 3. (**C**) Membranes were re-stripped and re-probed with anti β-tubulin antibody to evaluate equal loading. (**D**) Quantification of optical densitometry ratio between pPKA substrates and β-tubulin. *n* = 5. (**E**) Quantification of optical densitometry ratio between pY and β-tubulin. *n* = 3. Statistical significance comparing NL Caput vs. Lig Caput, Lig Caput vs. NL Cauda, and NL Cauda vs. Lig Cauda using an unpaired *t*-test in all graphs indicated; ns *p* > 0.05, * *p* < 0.05, ** *p* < 0.01.

It is well-established that cauda sperm incubated in capacitation-supporting media undergo cAMP-dependent phosphorylation of PKA substrates (pPKAs) followed by an increase in tyrosine phosphorylation (pY) [6]. On the other hand, immature caput sperm incubated in the same conditions fail to activate these pathways [11,12]. Confirming these results, when incubated in capacitation media, non-ligated caput sperm did not display an increase in pPKAs (Figure 4A, NL Caput). On the contrary, sperm recovered from ligated caput presented levels of pPKAs comparable in intensity to the pPKAs levels of mature cauda sperm (Figure 4A; Lig Caput). When we analyzed cauda sperm, we observed a slight decrease in pPKAs in sperm from ligated cauda, nevertheless it was not statistically significant (Figure 4A, NL and Lig Cauda). Regarding tyrosine phosphorylation (pY), ligated caput sperm displayed a slight increase in pY when compared to non-ligated caput sperm; however, the level of phosphorylation was minimal compared to pY levels of mature cauda sperm (Figure 4B). The pY levels of ligated cauda sperm were slightly reduced. Anti-tubulin Western blots were used as loading controls for optical densitometry analyses (Figure 4C–E). 

**Figure 5:**
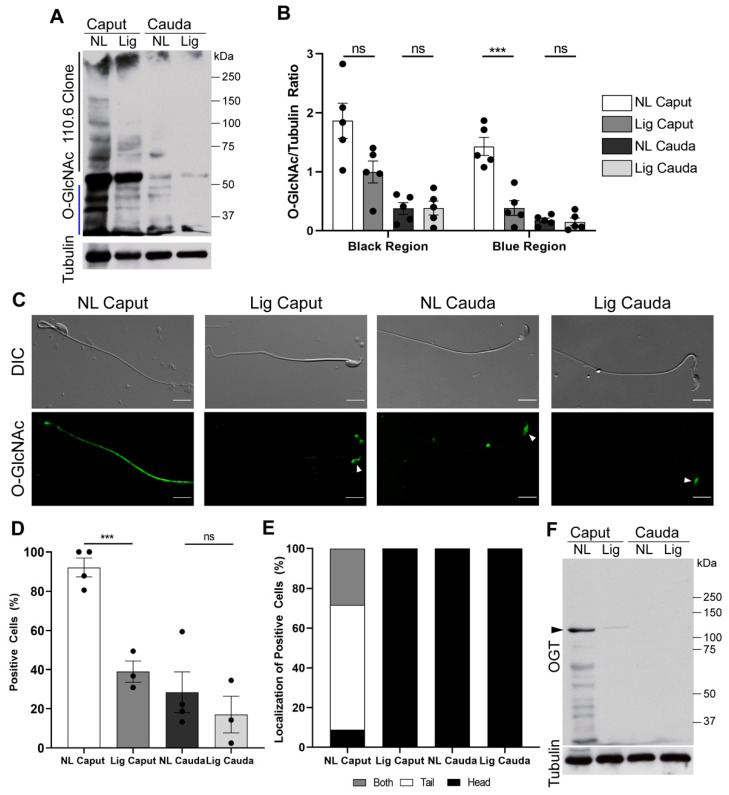
Changes in O-GlcNAcylated proteins after epididymal ligation. Sperm samples were collected from each experimental region 7-days post-ligation surgery. Samples were capacitated for 40 min prior to protein extraction and separation by SDS-PAGE. (**A**) Western blot of O-GlcNAcylated proteins (O-GlcNAc clone 110.6). Membranes were stripped and re-probed with anti β-tubulin as loading control. *n* = 5. (**B**) Quantification of optical densitometry ratio between O-GlcNAc and β-tubulin. Ratios were taken in two segments: area above 60 kDa (denoted by black line) and below 50 kDa (denoted by blue line). *n* = 5. (**C**) Localization of O-GlcNAcylated proteins (O-GlcNAc) in sperm by immunofluorescence. Upper panels, DIC microscopy images of sperm collected from NL Caput, Lig Caput, NL Cauda, and Lig Cauda. Lower panel, epifluorescence images of the localization of O-GlcNAc (green) in sperm collected from NL Caput, Lig Caput, NL Cauda, and Lig Cauda. White arrowheads indicate O-GlcNAc signal in the sperm head. Scale bars: 5 µm. *n* = 4. (**D**) Quantification of O-GlcNAc-positively stained sperm from each epididymal region. (**E**) Quantification of the cellular localization of O-GlcNac from the positively stained O-GlcNAc sperm. Independent experiments *n* = 4, individual sperm counted: NL Caput = 519, Lig Caput = 334, NL Cauda = 520, Lig Cauda = 520. (**F**) Western blot of O-GlcNAc Transferase (OGT, black arrowhead: 110 kDa). Membranes were stripped and re-probed with anti β-tubulin antibody as loading control. *n* = 3. Statistical significance comparing NL Caput vs. Lig Caput and NL Cauda vs. Lig Cauda using an unpaired *t*-test in graph indicated; ns, *p* > 0.05, *** *p* < 0.001.

As shown previously, non-ligated caput sperm had high levels of O-GlcNAcylation when compared to the ones observed for cauda sperm (Figure 5A, NL Caput vs. NL Cauda). On the other hand, when compared to sperm recovered from the non-ligated caput epididymis, ligated caput sperm depicted significantly lower levels of O-GlcNAcylation (Figure 5A, Lig Caput vs. NL Caput). Although most proteins showed a significant decrease in O-GlcNAc, a protein of ~ 55 KDa did not decrease its O-GlcNAc levels. Anti-tubulin Western blots were used as loading controls for optical densitometry of the indicated regions (Figure 5B). Previously, we have also shown that the enzyme responsible for O-GlcNAc post-translational modifications, OGT, is reduced during epididymal maturation [12]. Consistently, OGT is also reduced in ligated caput sperm (Figure 5C,F). Additionally, similar to our previous findings [12], the decrease in O-GlcNAc was observed by immunofluorescence. Overall, ligated caput sperm displayed reduced levels of O-GlcNAc comparable with those observed in cauda sperm (Figure 5C,D). Interestingly, O-GlcNAc in both ligated caput and cauda sperm is only found in the head region (Figure 5C,E) contrary to the tail localization observed in almost 90 % of non-ligated caput sperm (Figure 5C,E).

### 2.4. Ligated Caput Sperm Gain the Ability to Undergo Ionomycin-Induced Acrosome Reaction but Are Still Unable to Fertilize

**Figure 6:**
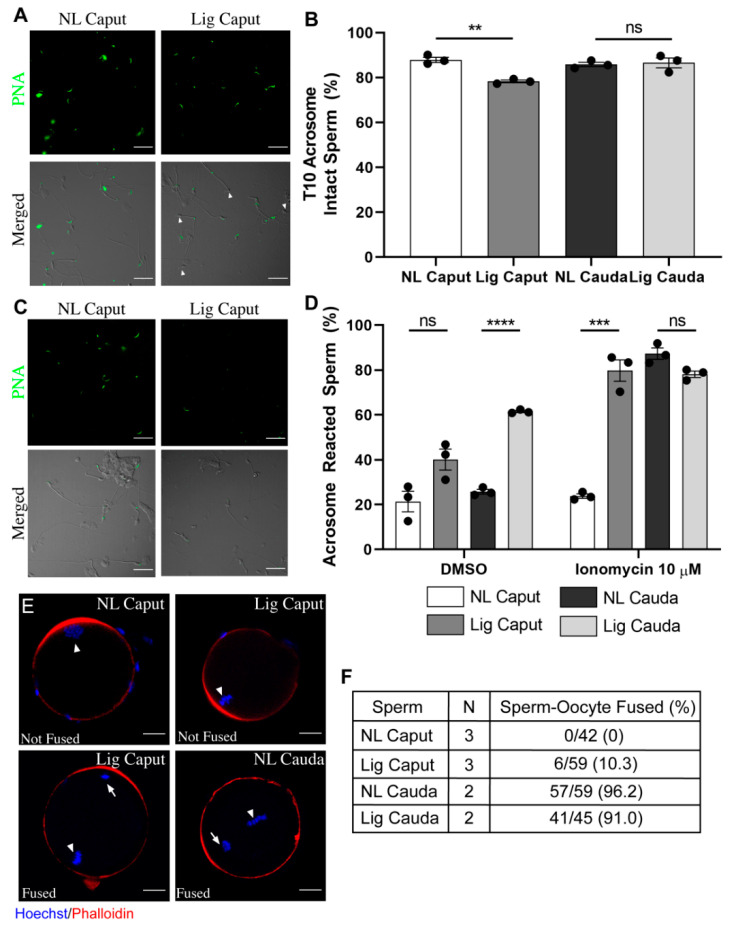
Effects of epididymal ligation on acrosome reaction and fusion. Sperm samples were recovered from each experimental region 7-days post-ligation surgery and placed into capacitation media. (**A**) Analysis of acrosomal status. Representative microscopy images of acrosomal status (PNA, green, upper panel) of NL Caput and Lig Caput after 10 min of capacitation (DIC, merged with PNA, lower panel). Scale bars: 10 µm. (**B**) Quantification of acrosome intact sperm at 10 min of capacitation. Independent experiments *n* = 3, individual sperm counted: NL Caput = 337, Lig Caput = 324, NL Cauda = 289, Lig Cauda = 256. (**C**) Analysis of acrosome reaction. After 60 min of capacitation, sperm samples were incubated with either DMSO or Ionomycin (10 µM) for 30 min to assess ability to acrosome react (as described in methods). Representative microscopy images of acrosome status (PNA, green, upper panel) of NL Caput and Lig Caput after incubation with ionomycin and DIC images merged with PNA fluorescence (lower panel). Scale bars: 10 µm. (**D**) Quantification of acrosome reacted sperm after incubation with either DMSO or Ionomycin. Independent experiments *n* = 3, individual sperm counted for each condition: NL Caput = 380, Lig Caput = 370, NL Cauda = 325, Lig Cauda = 390. (**E**) Capacitated sperm samples were co-incubated with zona-free MII oocytes for 60 min, followed by fixing and evaluation of fusion (as described in methods). Representative confocal microscopy images of Lig Caput (fused and not fused), NL Caput (not fused) and NL Cauda (fused) prospective zygotes co-stained with Phalloidin (red) to mark the subcortical actin cytoskeleton and Hoechst (blue) to mark female/male DNA. Arrowhead: female DNA, Arrow: male DNA. Scale bars: 25 µm. (**F**) Table depicting fusion assay results from all experimental conditions. Statistical significance comparing NL Caput vs. Lig Caput and NL Cauda vs. Lig Cauda using an unpaired *t*-test in all graphs indicated; ns *p* > 0.05, ** *p* < 0.01, *** *p* < 0.001, **** *p* < 0.0001.

As shown above, some of the signaling events associated with cauda sperm can be observed in sperm obtained from ligated caput epididymis. To further investigate the extent by which these sperm undergo capacitation, we analyzed their capacity to acrosome react, to fertilize in vitro and to fuse with zona-free eggs. Immediately upon suspension in capacitation-supporting media (T10), although in all conditions most sperm depicted intact acrosomes, a slight but significant increase in the spontaneous acrosome reaction, characterized by a reduction in PNA labeling, was observed in sperm recovered from ligated caput epididymis (Figure 6A,B). Once challenged with ionomycin, similar to cauda sperm, ~80 % of the ligated caput sperm population underwent the acrosome reaction. In contrast, ionomycin had no effect on the acrosome reaction of non-ligated caput sperm (Figure 6C,D).

Ultimately, capacitation renders spermatozoa able to fertilize an egg, form a zygote and start division. To test the potential of ligated caput to fertilize, sperm from the different epididymal regions were recovered in capacitation-supporting media and added to insemination droplets with cumulus enclosed or cumulus-free eggs. In both conditions, ligated and non-ligated cauda sperm fertilized, and the resultant zygote cleaved to form two-cell embryos (Table 1). In contrast, neither non-ligated nor ligated caput sperm were able to fertilize (Table 1). A few cumulus-free eggs exposed to caput sperm underwent cleavage; however, the most likely explanation is that a fraction of cumulus-free eggs suffered parthenogenic activation. Interestingly, when the fertilization assay was done with zona-free eggs, a small fraction of ligated caput sperm underwent fusion (Figure 6E,F). Nevertheless, those eggs with fused ligated caput sperm failed to cleave (Table 1).

## 3. Discussion

Before becoming fertilization competent, testicular mammalian sperm require to undergo epididymal maturation in males followed by capacitation in females. Epididymal maturation involves a series of biochemical and physiological changes that render the sperm able to move and to capacitate *in vivo* in the female tract or in vitro in appropriate media (reviewed in [1]). Epididymal maturation is a slow process that requires several days in which sperm transit through different regions of the epididymis. The complexity of this process has prevented development of in vitro systems that could successfully mimic the compartmentalized epididymal environments. Therefore, the study of sperm maturation relies on the comparison between sperm recovered from different regions of the epididymis (i.e., caput, corpus, cauda). Here, we show that ligation of the mouse epididymis at the distal caput can be used as a tool to investigate some aspects of sperm maturation. This approach has been used in the past in other species such as rabbits and rats [25,27,28,29]. Our results using a mouse system are consistent with these previous reports and show that a significant fraction of the immature sperm population acquire the capacity to move when retained in the caput epididymis for a period of at least 7 days. It has been proposed that epididymal maturation is triggered by a complex combination of exposure to a compartmentalized epididymal milieu and intrinsic sperm mechanisms depending on the total time spent in the epididymis [30]. In this regard, mouse sperm take 7–10 days to transit through the epididymis while maturing [31]. Our results indicate that caput epididymal ligation might be promoting intrinsic time-dependent sperm maturation events in caput sperm, and this technique could be a useful tool to dissect those processes that occur to sperm during maturation that are time- but not region-specific dependent.

Epididymal ligation did not affect sperm viability up to 7 days post-ligation, nor acrosomal integrity. The ligation procedure induced considerable tissue damage to a region in the caput epididymis as well as the enlargement of the remaining intact caput epididymal tubules. In addition, the epithelial composition of the caput epididymis changed after 7 days of ligation. This was evidenced by the reduction of AQP9 expression, a marker for principal cells, in the initial segment and caput epithelial cells. Principal cells are the main contributors of protein synthesis and secretions in the proximal epididymis [32,33,34]. Therefore, the observed reduction of AQP9 expression in the ligated initial segment and caput epididymis may indicate deficient principal cell function that could result in drastic changes in the luminal content to which sperm are exposed to in the post-ligation period and may contribute to the sperm gain in motility.

The ligation procedure did not have consequences in the overall cell composition of the cauda epididymal epithelium. It is worth mentioning that the epithelium of the corpus epididymis adjacent to the ligation site seemed to be affected, which would undermine analyses of corpus sperm. Additionally, longer periods of epididymal ligation (i.e., 10 and 14 days) were detrimental for sperm viability suggesting that the accumulation of sperm and lack of flow within the caput for extended periods of time induced sperm death. Altogether, these results support the utilization of 7-days epididymal ligation in the distal caput as a tool for the study of sperm maturation.

One of the hallmarks of epididymal maturation is the acquisition of sperm motility (reviewed in [1]). It is known that the machinery required for motility is assembled but maintained inhibited in caput sperm as demembranated caput sperm start to be motile when exposed to ATP or cAMP [35,36,37,38]. Here, we show that after 7 days of epidydimal ligation, caput sperm acquire progressive motility. It has been proposed that the acquisition of motility during epididymal maturation depends on inactivation of the sperm-specific alternative spliced isoform of the ser/thr phosphatase PP1γ known as PP1γ2 which becomes inactivated in cauda sperm [20]. This inactivation has consequences in the ability of cauda sperm to undergo capacitation-associated phosphorylation cascades. Consistently, caput sperm incubated in capacitation-supporting media (containing HCO_3_^−^ and BSA) failed to undergo phosphorylation of PKA substrates [12] and do not display the hallmark capacitation-associated increase in tyrosine phosphorylation [11,12]. In contrast, sperm from ligated caput undergo phosphorylation of PKA substrates to levels comparable to the ones observed for cauda sperm. These data indicate that PKA-dependent phosphorylation can occur in ligated caput sperm. However, differences in the molecular weight of several phosphorylated proteins observed with the anti-pPKAs antibody indicate that the phosphorylation pattern observed in ligated caput sperm is different to the one obtained for cauda sperm suggesting that ligation does not completely mimic the maturation process. Moreover, although we were able to observe a slight increase in tyrosine phosphorylation between ligated and non-ligated caput sperm, the level of phosphorylation does not reach to the one observed in cauda sperm. These results suggest that, in addition to the time of residence in the caput, interactions with other epididymal regions are necessary. 

Phosphorylation pathways are thought to be connected with another post-translational modification known as O-GlcNAc, this modification also occurs in ser and thr residues. Current hypotheses state that O-GlcNAc prevents phosphorylation on these residues, and, therefore, prevents activation of phosphorylation pathways. We have previously shown that protein O-GlcNAc decreases in mouse sperm during epididymal maturation [12]. Here we show that, similar to cauda sperm, sperm recovered from ligated caput also have a significant decrease in O-GlcNAc. As previously observed, the decrease in O-GlcNAc is mostly observed in the sperm tail in a localization reminiscent to the one observed for PKA [39]. Additionally, as previously reported [12], levels of OGT, the enzyme responsible for O-GlcNAc, were significantly reduced in ligated caput sperm. These results indicate that OGT and O-GlcNAcylation levels decrease over time during epididymal maturation independently of the epididymal milieu. The mechanisms that drive OGT degradation during epididymal maturation are still unknown. 

As shown, our results indicate that when sperm movement from the caput to other epididymal regions is restricted, those sperm acquire the ability to move progressively. Those sperm also undergo changes in signaling pathways such as the possibility to activate PKA-dependent phosphorylation and decrease in O-GlcNAc. Although these findings suggest that a fraction of the capacitation-associated pathways are possible without passage through other epididymal regions, to be fully mature, changes in other functional parameters are necessary. Physiological changes that occur to sperm during maturation include rearrangements of the sperm plasma membrane that give them the ability to acrosome react and to fuse with an MII oocyte. Consistent with previous reports in dogs and monkeys, immature non-ligated caput sperm were not able to acrosome react when challenged with the Ca^2+^ ionophore A_23187_ [40,41]. Here, we show that sperm recovered from non-ligated caput epididymis did not undergo acrosomal exocytosis when exposed to ionomycin, another Ca^2+^ ionophore able to induce high levels of acrosome reaction even in non-capacitated cauda sperm [42]. On the other hand, most sperm from ligated caput undergo the acrosome reaction when challenged with ionomycin. It was recently shown that AQPs expression in the different regions of the epididymal epithelium changes due to cryptorchidism in the dog [43]. The study of AQPs expression in the mouse caput epithelium after epididymal ligation and its relation to the acquisition of ability of sperm to acrosome react needs to be explored. Finally, our results evidence that caput epididymal ligation induces tissue damage of the caput epididymis. Then, we cannot discard that sperm in the ligated caput are exposed to higher levels of radical oxygen species (ROS). However, recent work from our laboratory indicates that, in the mouse, ROS generation does not influence cAMP-dependent pathways during sperm capacitation [44]. These results suggest that the increase in phosphorylation of PKA substrates observed in caput sperm upon epididymal ligation is not a consequence of increased ROS production. More work will be needed to further evaluate the role of ROS during this process. 

Although the ability to acrosome react is an essential component of epididymal maturation, to be considered complete, it is necessary that sperm gain fertilizing potential. To explore this possibility, we performed in vitro fertilization with ligated and non-ligated caput sperm using eggs in three different protocols: cumulus-enclosed, cumulus-free and zona-pellucida-free. Although ligated caput sperm can move progressively, they failed to undergo hyperactivation; therefore, it was not surprising to find that these sperm were not able to fertilize zona-intact eggs. When the assay was conducted with zona-free eggs, contrary to non-ligated caput sperm, a small fraction of the ligated caput sperm population was able to fuse. Fusibility of these sperm might be related to their ability to acrosome react. Interestingly, those eggs with fused ligated caput sperm were not able to cleave. These data are consistent with recent work indicating that caput sperm are not able to maintain early embryo development [19]. In this regard, it has been shown that during passage through the epididymis sperm acquire small non-coding RNAs that can affect fertilization and early embryo development [14,18].

Overall, our results indicate that ligated caput sperm can achieve some extent of maturation at the physiological level. Associated to the changes in motility, levels of O-GlcNAcylation were found to decrease in the ligated caput sperm as well as the levels of the enzyme OGT. Moreover, changes in phosphorylation of PKA substrates indicated that ligated caput sperm were able to activate pathways related to sperm capacitation. Despite these changes, sperm from ligated caput did not acquire the ability to fertilize in vitro. However, a small fraction of ligated caput sperm was capable of fusing with zona-free eggs. Altogether, these results show that the acquisition of motility and the capacity to undergo capacitation-associated changes in phosphorylation can occur in the absence of corpus or cauda epididymal secretions. 

Application of distal caput epididymal ligation has the potential to be useful to study certain aspects of sperm maturation beyond the comparison of sperm recovered from different regions of the epididymis. By applying this technique, we showed that molecular changes that induce sperm progressive motility can develop without transit and without secretions from corpus or cauda epididymis. Our results also indicate that ligated caput sperm cannot undergo full capacitation, and therefore cannot be considered completely mature without passage through all the different regions of the epididymis. These data highlight the complexity of epididymal maturation and the importance of epididymal transit to achieve full fertilizing potential. In this regard, it is well established that epithelial cells in different epididymal regions are transcriptionally and functionally different [45,46,47,48]. Further studies to evaluate functional changes in caput epithelial cells following ligation, and their role in sperm maturation are warranted.

## 4. Materials and Methods

**Animals.** Mouse sperm samples were collected from male CD1 retired breeders (Charles River Laboratories, Wilmington, MA, USA). Mouse oocytes were collected from 8–10-week-old super ovulated CD1 females (Charles River Laboratories, Wilmington, MA, USA). For superovulation, approximately 10 females per round of IVF were injected with 5 IU pregnant mare serum gonadotropin (PMSG) (Lee BioSolutions, cat # 493-10, Maryland Heights, MO, USA) and 5 IU human chorionic gonadotrophin (hCG) (Sigma, cat # CG5, St. Louis, MO, USA) 48 h later. Cumulus-Oocytes-Complexes (COCs) were collected 13 h post hCG injection. Animal care and use of experimental animals were conducted in accordance with specific guidelines and standards dictated by the Office of Laboratory Animal Welfare (OLAW) and approved by the Institutional Animal Use and Care Committee (IACUC), University of Massachusetts-Amherst (Protocol #2019-0008).

**Media.** Medium used for sperm capacitation was HEPES-based, modified Toyoda-Yokoyama-Hosi (m-TYH) medium consisting of 119.37 NaCl, 4.78 KCl, 1.19 KH_2_PO_4_, 1.19 MgSO_4_, 5.56 glucose, 1.71 CaCl_2_, 20 HEPES, 0.51 Na-pyruvate, 10 µg/mL gentamicin, 0.0006 % phenol red supplemented with 25 mM NaHCO_3_^−^ and 5 mg/mL of bovine serum albumin (BSA) (Sigma cat # A0281, St. Louis, MO, USA) at pH 7.2–7.4. Medium used for sperm fertilization assay and fusion assay was Toyoda-Yokoyama-Hosi (IVF-TYH) medium, consisting of 119.37 NaCl, 4.78 KCl, 1.19 KH_2_PO_4_, 1.19 MgSO_4_. 7 H_2_O, 5.56 glucose, 1.71 CaCl_2_.2 H_2_O, 25.1 mM NaHCO_3_^−^, 0.51 Na-pyruvate, 4 mg/mL BSA, 10 µg/mL gentamicin, 0.0006 % phenol red at pH 7.4 equilibrated with 5% CO_2_. This medium does not contain HEPES. Medium used for oocyte collection was Tyrodes’s lactate-HEPES (TL-HEPES), consisting of 114 mM NaCl, 3.22 mM KCl, 2.04 mM CaCl_2_.2 H_2_O, 0.35 mM NaH_2_PO_4_.2H_2_O, 0.49 mM MgCl_2_.6H_2_O, 2.02 mM NaHCO_3_^−^, 10 mM Lactic acid (sodium salt), and 10.1 mM HEPES at pH 7.4).

**Epididymal Ligation Surgery.** Male CD1 retired breeders were given analgesic meloxicam at a dose of 10 mg/kg via intraperitoneal (IP) injection five minutes prior to being anesthetized with 2–3% isoflurane via inhalation. Once the animal was non-responsive per IACUC protocol, a small 5 mm incision was made lateral to the penis on either ventral-left or ventral-right of the animal. Experiments were conducted ligating either the ventral-left or the ventral-right to account for asymmetrical differences in the epididymis. After dissecting through the skin and muscle layer, the caput region of the epididymis was identified, and two sutures were placed between the distal caput and proximal corpus. Animals received one suture to close the muscle layer and an additional two sutures to close the skin layer before being removed from isoflurane. Post-operative care was monitored for 2–14 days in relationship to collection time point.

**Sperm sample collection.** Time points of collection were conducted 2-, 5-, 7-, 10-, and 14-days post ligation surgery dependent on the experimental endpoint. Mice were euthanized by exposure to carbon dioxide (CO_2_). Then, the epididymides were dissected and the sperm samples were collected from the following regions: non-ligated caput (NL Caput), non-ligated cauda (NL Cauda), ligated caput (Lig Caput) and ligated cauda (Lig Cauda) (Figure S1). Each region was isolated in 500 µL of capacitating media. Sperm recovered from the NL and Lig Caput epididymides were obtained by squeezing the tissue in capacitating m-TYH or IVF-TYH media approximately 10–15 times. Sperm recovered from the NL and Lig Cauda epididymides were obtained by the “swim-out” method using the same capacitating media in a 10 min period. All samples were counted using a hemocytometer and adjusted to have a final concentration of 500,000–2,000,000 sperm per mL concentration for protein analysis. Samples used for fertilization assays and fusion assays were adjusted to have a final concentration of 100,000 sperm per fertilizing droplet (90 μL). Samples were capacitated for 40 min unless otherwise stated.

**Motility Assay and CASA Analysis.** Samples were analyzed at two time points of incubation: 10 and 40 min of capacitation. Sperm suspensions (35 μL) were loaded into a pre-warmed chamber slide (depth 100 μm) (Leja slide, Spectrum Technologies, Aurora, IL, USA) and placed on a microscope stage at 37 °C. Sperm motility was examined using the CEROS computer-assisted sperm analysis (CASA) system (Hamilton Thorne Research, Beverly, MA, USA). The default settings include the following: frames acquired: 90; frame rate 60 Hz; minimum cell size: 4 pixels; static head size: 0.13–2.43; static head intensity: 0.10–1.52; static head elongation: 5–100. At least 5 microscopy fields corresponding to the minimum of 500 sperm were analyzed for each treatment in each experiment.

**Immunocytochemistry (H&E) and immunofluorescence of the epididymis.** Epididymides were removed and fixed by immersion in 4% paraformaldehyde-lysine periodate (PLP) solution for 24 h at 4 °C, then washed in PBS, and stored at 4 °C in PBS containing 0.02% sodium azide. PLP-fixed epididymal slices were cryoprotected in 30% Sucrose/PBS for at least 48 h at room temperature. They were embedded in Tissue-Tek OCT compound 4583 (Sakura Finetek, Torrance, CA) and frozen at −20 °C. Sections were cut at 5 µm using a Leica CM3050S cryostat (Leica, Wetzlar, Germany), and picked up onto Fisherbrand Superfrost Plus microscope slides (Fisher Scientific, Pittsburg, PA, USA). H&E staining (Millipore, Sigma, Burlington, MA, USA) was performed for overall morphology and the sections were scanned by using a digital slide scanner, NanoZoomer 2.0RS (Hamamatsu, Japan). For AQP9 and V-ATPase labeling: epididymal sections were hydrated in PBS for 15 min, and treated with sodium dodecyl sulfate (SDS) for 4 min. Slides were blocked in 1% BSA, and incubated with the primary antibody at 4 °C overnight, and with the secondary antibody for 1 h at room temperature (RT). The primary antibodies used were chicken polyclonal anti-A subunit V-ATPase (0.3 μg/mL) and rabbit polyclonal anti-aquaporin 9 (AQP9, 0.5 μg/mL) made and purified in Breton’s laboratory. The corresponding secondary antibodies (Jackson Immunoresearch Laboratories Inc., West Grove, PA, USA) were donkey anti-chicken IgG conjugated to Alexa488 (30 μg/mL, cat. no.: 703-545-155) and donkey anti-rabbit IgG conjugated to indocarbocyanine (Cy3) (7.5 μg/mL cat. no.: 711-165-152). All antibodies were diluted in DAKO medium (Dako, Carpinteria, CA, USA). Slides were mounted with SlowFade Diamond Antifade Mount medium (Thermo Fisher Scientific, Waltham, MA, USA) containing the DNA marker DAPI (Vector Laboratories, Burlingame, CA, USA), and were examined using the 90i Nikon microscope.

**Viability Assay.** After collection, sperm samples were capacitated for 30 min in capacitating m-TYH. Then the addition of Hoescht 33258 (Molecular Probes, H-1398) at a concentration of 1 μg/μL was added to the incubation for an additional 10 min. Once finished, samples were centrifuged at 800× *g* for 5 min, washed with filtered PBS and air-dried on Poly-L-Lysine-coated glass coverslips, followed by mounting using VectaShield (H-1000, Vector Laboratories, Burlingame, CA, USA) on slides. Images were taken using a 60X objective (Nikon, PlanApo, NA 1.49) on a fluorescence microscope (Nikon Eclipse T300). Conditions for analysis were the following: uptake of fluorescence probe indicative of dead sperm. Differential interference contract (DIC) images were taken in parallel and served as a control for live sperm. A minimum of 200 sperm were analyzed per condition per experiment.

**SDS/PAGE and Western Blotting.** After collection, sperm samples were capacitated for 40 min in full capacitation m-TYH media and then extracted for Western blot analysis as previously described [12]. Western blotting was performed using the following antibodies: anti-pPKA substrates monoclonal antibody (clone 100G7) diluted 1:10,000 (Cell Signaling, # 9624, Danvers, MA, USA); monoclonal anti-pY antibody (clone 4G10) diluted 1:10,000 (Millipore, cat # 05-321, Burlington, MA); O-GlcNAc monoclonal antibody (clone 110.6) diluted 1:2000 (Cell Signaling, cat # 9875, Danvers, MA, USA); OGT polyclonal antibody 1:1000 (Cell Signaling, cat # 5368, Danvers, MA, USA). Optical densitometry analysis was performed in all blots using the Fiji software. Each lane was normalized to tubulin. For pPKA-substrates analyses, the entirety of the lane was compared between conditions. For pY, the region below the hexokinase (110 kDa) was compared between conditions. For O-GlcNAc, two regions were compared as indicated in the figure, the upper region (>56 kDa, indicated with a black line) and lower region (<50 kDA, indicated with a blue line) for comparison between conditions. For OGT, the specific band at 110 kDa was used to compare between conditions.

**Sperm Immunolocalization.** After collection of sperm samples as described above, samples were fixed in 4% paraformaldehyde (EMS, Hatfield, MA) in PBS for 10 min at room temperature. Sperm samples were centrifuged at 800× *g* for 5 min, washed with PBS, and air-dried on Poly-L-Lysine-coated glass slides. For staining with O-GlcNAc or OGT, samples were processed as previously described [12]. Negative controls were run in parallel by incubation with secondary antibody alone. Images were taken using a 60X objective (Nikon, PlanApo, NA 1.49) on a fluorescence microscope (Nikon Eclipse T300). Differential interference contrast (DIC) images were taken in parallel and served as control for sperm morphology. A minimum of 100 sperm were analyzed per condition per experiment.

**Acrosome Reaction Assay.** After collection, sperm samples were incubated in capacitating m-TYH media. Immediately, 100 μL was taken from each condition (T10) and centrifuged at 800× g for 5 min at room temperature, washed with PBS and fixed with 4% paraformaldehyde (EMS, Hatfield, MA) for 10 min at room temperature. The suspension was then centrifuged at 800× g for 5 min and washed twice in PBS. Samples were stored at 4 °C during the remaining capacitation time of other samples. After 40 min of capacitation, each sperm sample from each region was divided and subjected to either DMSO or Ionomycin (20 μM) (Enzo, cat #ALX-450-007, Farmingdale, NY, USA). After an additional incubation of 30 min, the samples were processed in the same manner as the T10 samples. All samples were left to air dry on Poly-L-Lysine-coated coverslips, followed by permeabilization with 100% methanol for 30 s and washed with filtered PBS three times. Sperm were incubated with Alexa 488-conjugated peanut agglutinin (PNA) 10 μg/mL (Molecular Probes, cat #L-21409, Eugene, OR, USA) for 1 h at room temperature in a humidified chamber and then washed three times with PBS and mounted as described above. Images were taken using a 60X objective (Nikon, PlanApo, NA 1.49) on a fluorescence microscope (Nikon Eclipse T300). Differential interference contrast (DIC) images were taken in parallel and served as control for sperm morphology.

**Fusion Assay and analysis.** For Zona-Free IVF: cumulus-oocyte-complexes (COCs) were collected 13-h post hCG. Removal of the cumulus cells, COCs were placed in a 30 μL droplet of hyaluronidase for 2–5 min, then proceeded by washing in IVF-TYH media. Followed by removal of the zona pellucida, oocytes were placed in a 10 μL droplet of acid tyrode’s solution for 30–60 s then washed in IVF-TYH media. Oocytes were placed in a 90 μL droplet dish prior to fertilization and allowed to rest for 30 min in the incubator at 37 °C, 5% CO_2_. After collection of sperm as described above, the insemination droplet was inseminated with approximately 10,000 sperm per 90 μL droplet for 1 h. Oocytes were then washed thoroughly in IVF-TYH to remove extra sperm and placed into 4% PFA for 20 min at room temperature, followed by three washes with PBS. Samples were then permeabilized with 0.5% Triton X-100 for 20 min at RT, followed by three washes with 0.1% Triton X-100 in PBS. Oocytes were then incubated for 1 h at RT with 10% normal goat serum and incubated overnight in the presence of 6.6 μM phalloidin (Invitrogen, cat #A22287, Carlsbad, CA, USA) and Hoechst 33342 (Fisher Scientific, cat #H3570, Waltham, MA, USA). Images were taken using a 40X (Plan Fluor, NA 1.3) objective in a Nikon confocal microscope. Fused embryos were considered when Hoechst-stained sperm heads were visualized within the plasma membrane of the oocyte during 3D-analysis.

**In vitro fertilization (IVF) assay.** For Cumulus-intact Standard IVF: cumulus-oocyte-complexes (COCs) were collected 13 h post hCG and washed with IVF-TYH prior to being placed in the insemination droplet. After collection of sperm as described above, the insemination droplet was inseminated with approximately 100,000 sperm per 90 μL droplet. After 4 h, oocytes were washed in IVF-TYH and allowed to culture for 20 h. The following day, fertilization was investigated by visualization of 2-cell cleavage. For Cumulus-Free IVF: cumulus-oocyte-complexes (COCs) were collected 13 h post hCG. For removal of the cumulus cells, COCs were placed in a 30 μL droplet of hyaluronidase for 2–5 min, then proceeded by washing in IVF-TYH media. Oocytes were placed in a 90 μL droplet dish prior to fertilization and allowed to rest for 30 min in the incubator at 37 °C, 5% CO_2._ After collection of sperm as described above, the insemination droplet was inseminated with approximately 100,000 sperm per 90 μL droplet. After 4 h, oocytes were washed in IVF-TYH and allowed to culture for 20 h. The following day, fertilization was assessed by visualization of 2-cell cleavage. For Zona-Free IVF: cumulus-oocyte-complexes (COCs) were collected 13-h post hCG. Removal of the cumulus cells, COCs were placed in a 30 μL droplet of hyaluronidase for 2–5 min, then proceeded by washing in IVF-TYH media. For removal of the zona pellucida, oocytes were placed in a 10 μL droplet of acid tyrode’s solution for 30–60 s then washed in IVF-TYH media. Oocytes were placed in a 90 μL droplet dish prior to fertilization and allowed to rest for 30 min in the incubator at 37 °C, 5% CO_2_. After collection of sperm as described above, the insemination droplet was inseminated with approximately 10,000 sperm per 90 μL droplet. After 1 h, oocytes were washed in IVF-TYH and cultured for 20 h. The following day, cleavage was assessed by visualization of 2-cell cleavage.

**Statistical Analysis.** Data from all studies were analyzed using PRISM 8 Graph Pad software (https://www.graphpad.com/scientific-software/prism/). Data expressed as the mean ± S.E.M with individual experimental values represented by dots. For Caput Data: non-paired *t*-tests, or when applicable Sidak–Bonferroni *t*-tests were performed between NL Caput and Lig Caput. For Cauda Data: non-paired *t*-tests, or when applicable Sidak–Bonferroni *t*-tests were performed between NL Cauda and Lig Cauda. Statistical significances are indicated in the figure legends.

## Figures and Tables

**Table 1:** Cumulus-enclosed Standard, Cumulus-Free and Zona-Free in vitro fertilization assays. Sperm samples were recovered from each experimental condition and placed into capacitation media. Cumulus-enclosed Standard IVF: samples were co-incubated with cumulus-oocyte-complexes (COCs) for 4 h, before removal from insemination droplet. Fertilization was evaluated 24 h later by visualization of 2-cell embryos. *n* = 3. Cumulus-Free IVF: prior to insemination, COCs were incubated with hyaluronidase to remove cumulus cells as described in methods. Samples were then co-incubated with MII-oocytes for 60 min, removed from the insemination droplet and fertilization was determined by visualization of 2-cell stage embryos. *n* = 3. Zona-Free IVF: prior to insemination, MII-oocytes were incubated with Tyrode’s acid to remove zona pellucida as described in methods. Samples were then co-incubated with MII-oocytes for 60 min, removed from the insemination droplet and fertilization was determined by visualization of 2-cell embryos. *n* = 9.

Type of IVF	Sperm Collection Region	N	Fertilization/Total (%)
Cumulus-enclosed Standard IVF	NL Caput	3	0/185 (0)
	Lig Caput	3	0/169 (0)
	NL Cauda	3	199/206 (96.5)
	Lig Cauda	3	120/136 (88.2)
Cumulus-Free IVF	NL Caput	3	4/55 (4.2)
	Lig Caput	3	3/53 (3.2)
	NL Cauda	3	26/50 (62.2)
	Lig Cauda	3	22/43 (54.8)
Zona-Free IVF	NL Caput	9	0/62 (0)
	Lig Caput	9	0/72 (0)
	NL Cauda	9	75/75 (100)
	Lig Cauda	9	41/41 (100)

## Supplementary Materials

The following are available online at https://www.mdpi.com/article/10.3390/ijms221910241/s1.
