# Supplementary figures and images for: Caput Ligation Renders Immature Mouse Sperm Motile and Capable to Undergo cAMP-Dependent Phosphorylation
Source: Int J Mol Sci. 2021 Sep 23;22(19):10241. doi: 10.3390/ijms221910241 (PMC8549708; doi:10.3390/ijms221910241)

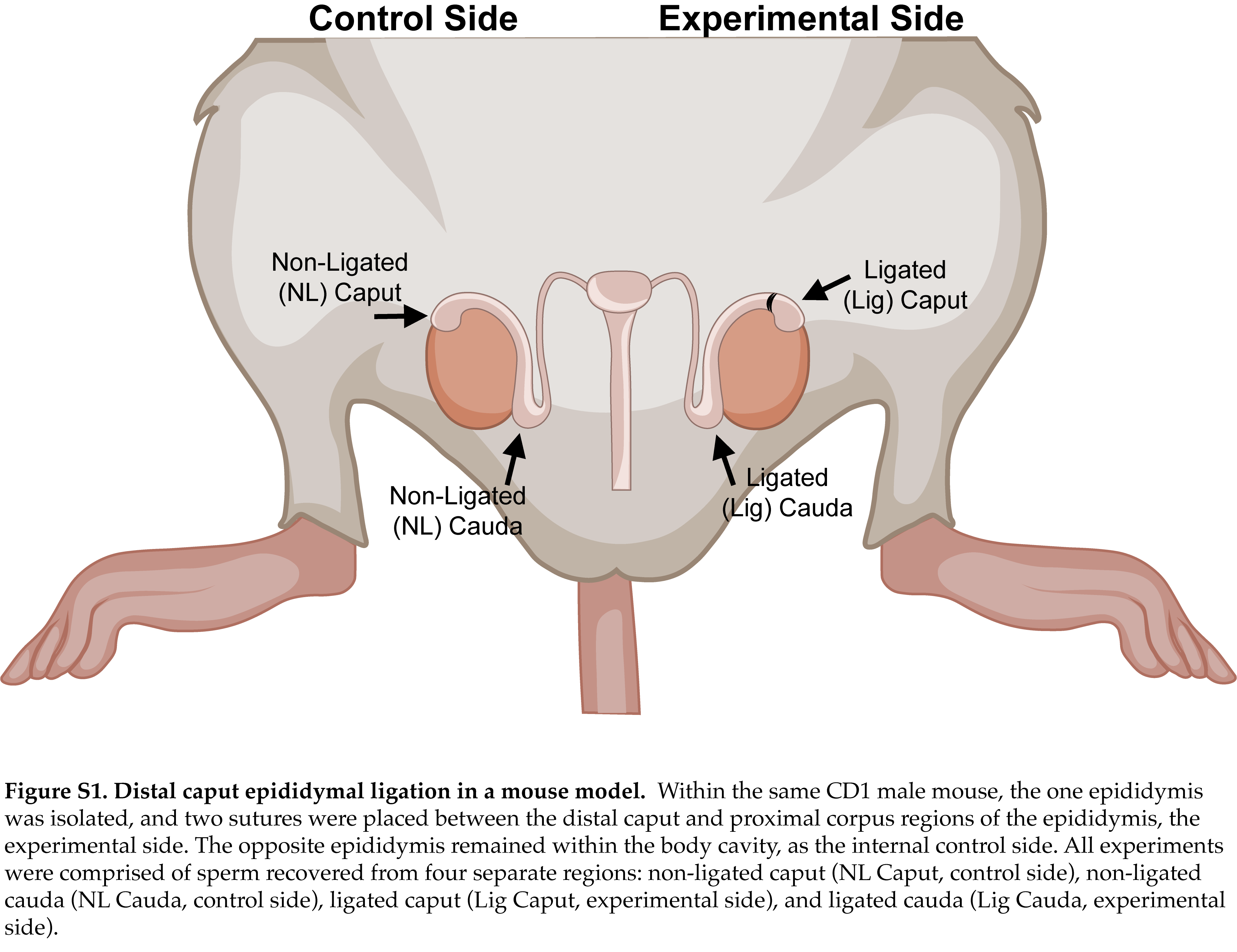

Supplement: Supplementary file 1 [file ijms-22-10241-s001.zip › ijms-1361022-supplementary/DT_Ligation_SFigure 1.tif]

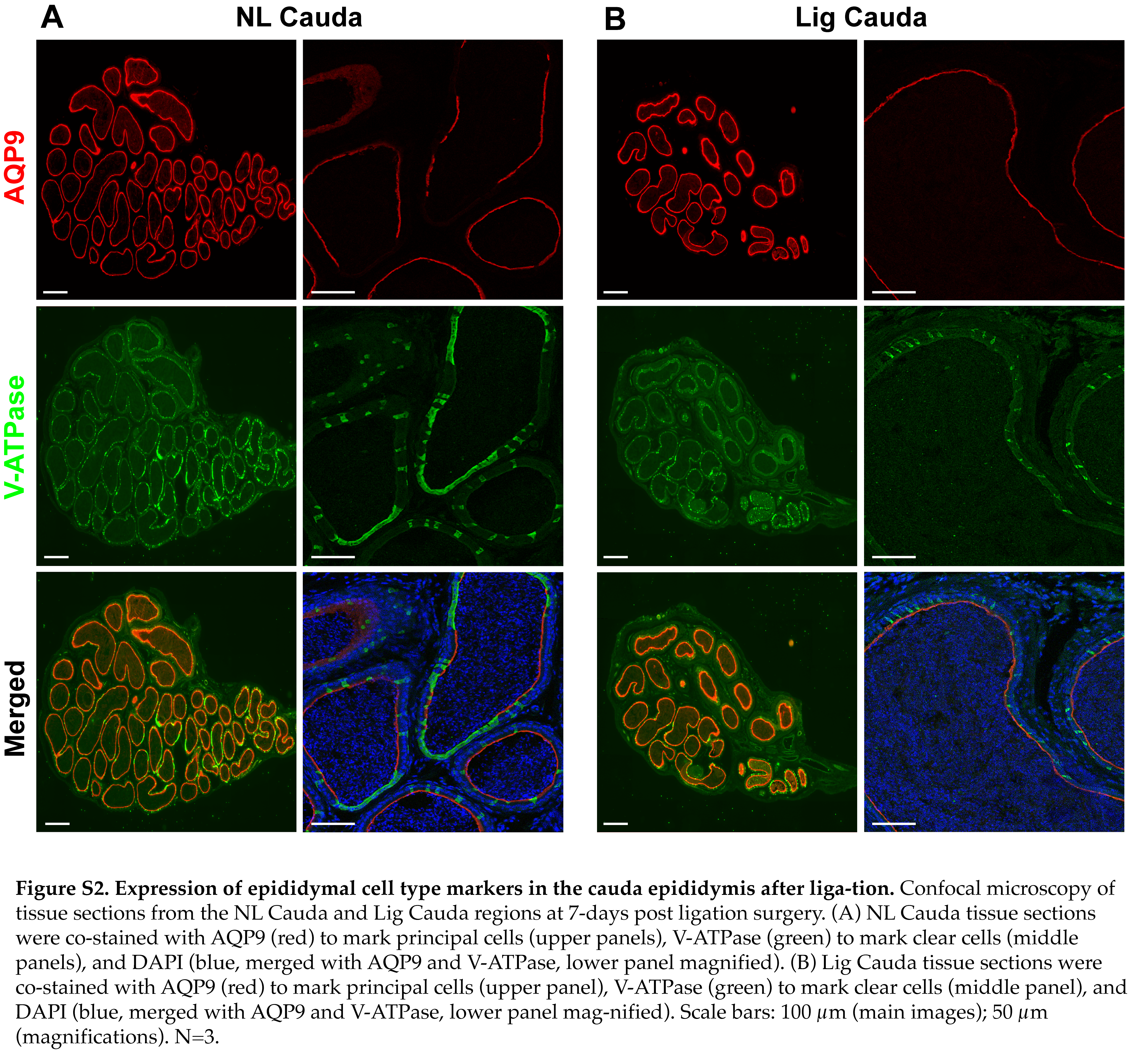

Supplement: Supplementary file 1 [file ijms-22-10241-s001.zip › ijms-1361022-supplementary/DT_Ligation_SFigure 2.tif]
